# On Farm Evaluation of a Novel Mechanical Cervical Dislocation Device for Poultry

**DOI:** 10.3390/ani8010010

**Published:** 2018-01-10

**Authors:** Jessica E. Martin, Victoria Sandilands, Julian Sparrey, Laurence Baker, Dorothy E. F. McKeegan

**Affiliations:** 1 The Royal (Dick) School of Veterinary Studies and The Roslin Institute, Easter Bush Campus, The University of Edinburgh, Edinburgh EH25 9RG, UK; 2 Monogastric Science Research Centre, Animal and Veterinary Sciences Research Group, SRUC, Auchincruive Campus, Ayr KA6 5HW, UK; vicky.sandilands@sruc.ac.uk (V.S.); laurence.baker@sruc.ac.uk (L.B.); 3 Livetec Systems Ltd., Building 52, Wrest Park, Silsoe, Bedford MK45 4HS, UK; sparrey@livetecsystems.co.uk; 4 Institute of Biodiversity, Animal Health and Comparative Medicine, College of Medical, Veterinary & Life Sciences, University of Glasgow, Glasgow G61 1QH, UK; dorothy.mcKeegan@glasgow.ac.uk

**Keywords:** killing, poultry, cervical dislocation, reflexes, post-mortem, animal welfare

## Abstract

**Simple Summary:**

Large numbers of poultry are killed on farm (usually because they are ill or injured) and we have a responsibility to ensure that the methods used have minimal welfare impact. The traditional method of manual cervical dislocation (i.e., “necking” by hand), has been subject to welfare concerns and has recently been restricted by law in Europe, in terms of the number of birds that can be killed with this method per day. Alternative methods need to be developed and these must be humane, practical and reliable in commercial settings. We evaluated the performance and reliability of a novel mechanical cervical dislocation device in comparison with the traditional manual method. We tested the performance of multiple users of each method in commercial laying hen and broiler farm settings. The novel mechanical method was outperformed by the traditional manual method, and there were some issues with training, dependent on the stockworkers’ technique and experience. The results show that while the novel method has the potential to improve welfare, it requires further refinement and training optimization in order to provide a viable alternative to manual cervical dislocation across the poultry industry.

**Abstract:**

Urgent development of alternative on-farm killing methods for poultry is required following the number restrictions placed on the use of traditional manual cervical dislocation by European Legislation (EU 1099/2009). Alternatives must be proven to be humane and, crucially, practical in commercial settings with multiple users. We assessed the performance and reliability of a novel mechanical cervical dislocation device (NMCD) compared to the traditional manual cervical dislocation (MCD) method. NMCD was based on a novel device consisting of a thin supportive glove and two moveable metal finger inserts designed to aid the twisting motion of cervical dislocation. We employed a 2 × 2 factorial design, with a total of eight stockworkers from broiler and layer units (four per farm) each killing 70 birds per method. A successful kill performance was defined as immediate absence of rhythmic breathing and nictitating membrane reflex; a detectable gap in the vertebrae and only one kill attempt (i.e., one stretch and twist motion). The mean stockworker kill performance was significantly higher for MCD (98.4 ± 0.5%) compared to NMCD (81.6 ± 1.8%). However, the MCD technique normally used by the stockworkers (based previous in-house training received) affected the performance of NMCD and was confounded by unit type (broilers), with the majority of broiler stockworkers trained in a non-standard technique, making adaption to the NMCD more difficult. The consistency of trauma induced by the killing methods (based on several post-mortem parameters) was higher with NMCD demonstrated by “gold standard” trauma achieved in 30.2% of birds, compared to 11.4% for MCD (e.g., dislocation higher up the cervical region of the spine i.e., between vertebrae C0–C1, ≥1 carotid arteries severed), suggesting it has the potential to improve welfare at killing. However, the results also suggest that the NMCD method requires further refinement and training optimization in order for it to be acceptable as an alternative across poultry industry, irrespective of previous MCD technique and training.

## 1. Introduction

Killing of poultry on farm is a necessity due to the need to rapidly cull individual injured and sick birds, and to a lesser extent for stock management [1]. The methods used to cull small numbers of birds on-farm are different from slaughter [2,3,4] or emergency on farm-killing of whole flocks [4,5,6] and have been under heavy scientific scrutiny for the last decade. It may be estimated that the number of chickens routinely killed on farm is up to 9 billion per annum globally (based on mortality rates (including “found dead”) ranging from 1 to 15% of a total population of 60 billion) making the methods used a significant welfare concern. 

The primary methods employed by the industry and backyard poultry keepers to dispatch individual birds on farm are manual cervical dislocation (designed to cause death by cerebral ischaemia and extensive damage to the spinal cord and brainstem [4,7,8,9,10,11,12,13]) and application of percussive devices (designed to cause extensive direct brain damage, resulting in brain death [1,9,10,11,14]). Welfare concerns have been raised about mechanical cervical dislocation in particular, in both poultry [9,15] and other species [7,16,17] since it has been suggested that animals may be conscious for a significant period post-application [9,11,18]. However, more recent work has questioned this [7,12,13] and suggests that the results of earlier studies did not correctly reflect the performance and efficacy of well-performed manual cervical dislocation. It has been noted that there is high variability in the application of any type of cervical dislocation by different relevant groups (e.g., poultry stockworkers, veterinarians, trained slaughtermen) [1,14].

Since January 2013 the use of manual cervical dislocation (MCD) as a killing method for poultry on-farm has been heavily restricted through EU legislation, Regulation (EC) No. 1099/2009 On the Protection of Animals at the Time of Killing [19]. Specifically, MCD can only be used by each person on 70 birds per day weighing less than 3 kg. Several mechanical devices have been developed in order to provide alternative methods for dispatching birds on-farm over the last ten years (e.g., CASH Poultry Killer, Turkey Euthanasia Device) [3,9,10,20,21] and these could theoretically be used in place of MCD, however none have been enthusiastically adopted across the commercial industry or by small poultry keepers. Mechanical cervical dislocation is when the operator uses a tool to perform the method (e.g., killing cone, burdizzo, pliers [1,3]). Prior to the legislation change, these methods were primarily used for large birds (e.g., turkeys and ducks), however, following 2013 mechanical methods have been more widely used following the weight and frequency restrictions applied to MCD [22]. The success of a killing device can be defined in three ways; welfare impact (humaneness), reliability and practicality. Humaneness and reliability are important for determining the suitability of a device; however, assessing practicality and user-reliability is also an essential part of assessment if industry and wider uptake is a realistic goal. 

We developed a novel mechanical cervical dislocation device (NMCD) and evaluated it in laboratory settings [12,13] for dispatching individual broilers and layers. The device consists of a glove and two moveable metal finger inserts designed to aid the twisting motion of cervical dislocation and it shows substantial potential as a humane and reliable mechanical method when used by a single operator under controlled conditions. For example, analysis of electoencephalogarm (EEG) activity during killing of lightly anaesthetized birds demonstrated that the birds were unconscious within a mean of 3.1 s post device application, but in the cases where the device behaved optimally this duration further decreased to between 1.5–2.3 s. Induction of rapid loss of consciousness post device application was also supported by measurements of loss of reflexes and onset of death-associated behaviors [12,13,22]. 

In commercial settings, it is usually not feasible to record EEG signals to confirm unconsciousness and death [23], therefore the presence/absence of reflexes are used to determine loss of consciousness (e.g., jaw tone) [9,23,24,25] and brain death (e.g., pupillary reflex, nictitating membrane reflex) [2,8,24,26,27,28,29]. Post-mortem analysis has also been used to effectively assess gross anatomical damage caused by killing methods, and are useful in welfare assessment where specific damage can be associated with likely rapid loss of consciousness [13,30]. Optimal application of either manual or mechanical cervical dislocation should result in separation of the skull from the vertebral column at C0–C1 and sever both carotid arteries, without damaging the exterior of the bird [3,13]. The aim of this experiment was to determine the efficacy of our novel mechanical cervical dislocation (NMCD) device in a commercial setting, compared to traditional manual cervical dislocation (MCD). To obtain information that was as representative as possible of the wider poultry industry, we examined the performance of each method in multiple poultry stockworkers and examined application of the methods in both broiler and layer chickens.

## 2. Materials and Methods

A total of 1120 birds (*Gallus gallus domesticus*) were used across a layer farm and a broiler farm; 560 laying hens (58 weeks old, Hy-Line strain) and 560 mixed-sex slaughter-age broilers (38 days old, Ross 308 strain). The birds were kept in commercial conditions until killing occurred. The layer hens were housed in enriched colony cages (Tecno Cages^®^, Tecno Poultry Equipment Spa, EU); 80 birds per colony. The broilers were floor housed in an open-plan shed with deep litter (wood-shavings). All birds had ab libitum access to food and water. The experiment was performed under UK Home Office licence authority via Project and Personal licenses and underwent review and approval by SRUC’s ethical review body. 

The trial had a 2 × 2 factorial design, with a total of eight stockworkers (four per farm) assessed for their performance with the NMCD device compared to MCD. The NMCD device has been described previously [12,13,22]. Briefly, it was designed to mirror the technique of manual cervical dislocation, but adding a mechanical component. The device consisted of a fabric supportive glove (SHOWA 370 Multipurpose Stable Glove^TM^, UK) designed to support the wrist and hand and two moveable metal finger inserts, which were designed to fit around the bird’s head to create a secure grip, and to move independently in order to allow adjustment for different sizes of birds. The rounded shape of the metal fingers was designed to aid the twisting motion of cervical dislocation [1,13] required to dislocate the bird’s neck. The blunt edge between the two metal fingers (protruding <1 mm from the fleshy area of skin between the index and middle fingers) provided a hard edge to force between the back of the bird’s head and the top of the neck, designed to focalize the force at C0–C1 when the method was applied.

The MCD technique used was dependent on the stockworker’s previous training and standard operating procedures at each unit, and did not always follow HSA’s guidelines [3]. Variation in the technique related to how the bird’s head was held in the operator’s palm, either with the neck between the index and middle finger (V-shaped or “V” technique) or the neck held between the thumb and the index finger (ring-shaped or “R” technique). In general, MCD was performed in one swift movement with the operator pulling down on the bird’s head, stretching the neck, while rotating the bird’s head upwards into the back of the neck. The eight male stockworkers selected for the trial were experienced in performing MCD on a regular basis and were deemed competent by their respective on-site farm managers. Biometric measures of all stockworkers were recorded (e.g., hand span, hand length, arm length, height and weight), as well as their handedness and MCD technique. 

On each farm 70 birds were killed with each method (NMCD/MCD) per stockworker (*n* = 4) across two days. Each stockworker performed both killing methods (NMCD/MCD) within a day, with kill order, session (morning/afternoon) and killing method systematically randomized. One stockworker performed one killing method at a time, with another assisting by collecting birds. The work adhered to 3Rs principles as all birds were destined for slaughter (broilers) or culling (end of lay hens). All birds were weighed and identified with a numbered leg tag prior to killing. 

**Figure 1:**
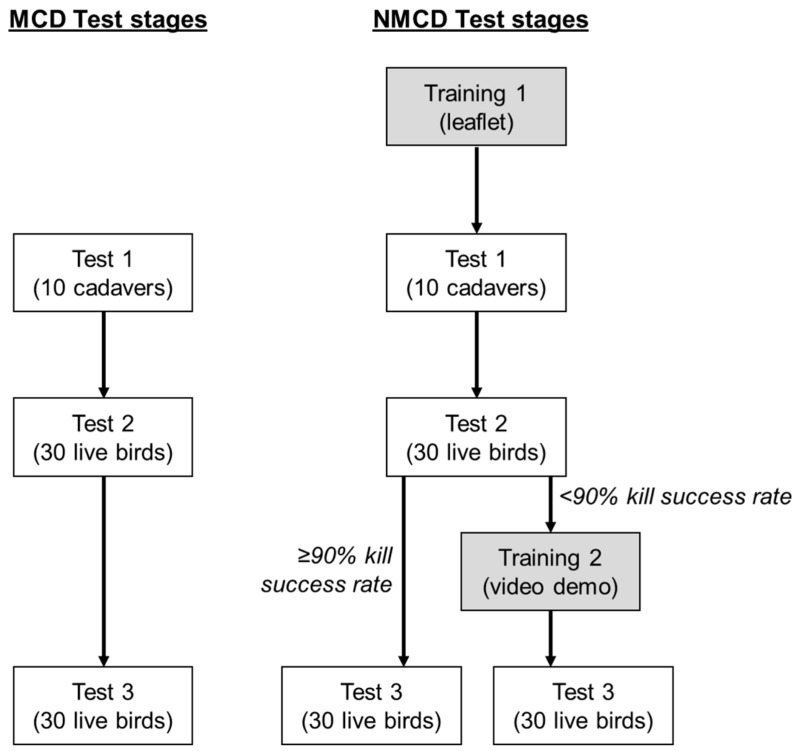
Flow chart of the experimental procedure for each killing method.

In order to assess the training requirements for NMCD and the kill efficacy of each killing method as applied by each stockworker, the 70 birds per killing method were sub-divided into three test stages in order to minimize the risk to bird welfare: Test 1—applied to 10 cadavers; Test 2—applied to 30 live conscious birds; and Test 3—applied to 30 live conscious birds (Figure 1). In Test 1, birds were euthanised immediately prior to testing in the predetermined test order with an intravenous overdose of sodium pentobarbital (Euthatal, Merial Animal Health Ltd., Essex, UK; 1 mL per kg). There was no maximum time for completion of each test, but the time for completion of each was recorded. The killing rate of birds within each test was not controlled in an attempt to reduce any stress on the stockworkers and to allow them to perform the killing methods at comfortable rates. Between tests, the stockworkers took standardized 5 min breaks. No training relating to MCD was provided prior to testing and the three tests were performed consecutively for both killing methods. As NMCD was a novel method, and one aim was to gauge the training required to apply it successfully, two training stages were included. Before Test 1, compulsory training was provided (Figure 1, Training 1), and further training was provided depending on performance in Test 2 (Training 2). Training 1 involved providing the stockworker with a leaflet which simulated the instruction manual which would accompany the device if purchased. Each stockworker was given time to read the leaflet and try on the various sizes of the gloved device (small/medium/large) in order to select the appropriate size. The stockworker was then given 10 cadavers to perform the NMCD method on. This allowed the stockworker to become accustomed (e.g., by adjusting hand grip) to the device without compromising bird welfare, although there was a lack of muscle tone compared to live birds. Kill efficacy (sufficient trauma to cadaver e.g., cervical dislocation) was recorded for each bird and at the end of Test 1 the stockworker was asked if he was comfortable to continue to the next test (on live birds). If yes, then following the 5 min break the stockworker continued on to Test 2. If no, the tests for NMCD were halted for that stockworker and no further birds were killed by that method. In Test 2, each stockworker was given 30 live and conscious birds to kill. If the overall kill efficacy (see below) was less than 90%, training stage 2 was provided before progressing to Test 3. At the end of Test 2 the stockworker was asked if he was comfortable to continue to Test 3. If yes, then following a 5 min break the stockworker continued. If no, the tests for NMCD were halted for that stockworker and no further birds were killed by that method. For each method, all three tests were performed consecutively within one session (morning (AM) or afternoon (PM)).

Kill efficacy was determined by a trained experienced poultry technician immediately post application of a method for each bird by assessment of four parameters: (1) cessation of rhythmic breathing; (2) absence of nictitating membrane; (3) a gap in the neck and (4) observation of only one kill attempt (i.e., one stretch and twist action). Multiple attempts (i.e., multiple pulls) were recorded as a fail, even if they resulted in the death of the bird. In Test 1, kill efficacy on cadaver birds was established by the confirmation of a gap between two cervical vertebra via externally palpation of the neck and no greater than one attempt to achieve this. In Tests 2 and 3, if the birds did not display signs of death rapidly post-application, they were immediately emergency euthanised with MCD by the poultry technician. If at any point during the tests the stockworkers became uncomfortable and did not want to continue, the test was halted and for NMCD additional training was offered, depending on the training stage completed.

A post-mortem examination was performed on every bird immediately after the application of the killing method in Test 1 or after confirmation of death in Tests 2 and 3. Binary yes/no measures were recorded for observations of skin tears, external blood loss, subcutaneous hematoma, dislocation of the neck, vertebra damage (e.g., intra-vertebra dislocation/break), and whether the spinal cord was severed. The vertebrae between which cervical dislocation had occurred was recorded (e.g., C0–C1, C1–C2 etc.), as well as the length (cm) of the gap between the dislocated cervical vertebra. Dislocation level was then converted to a number (C0–C1 = 1, etc.) for analysis. The number of carotid arteries severed was observed during the post-mortem and recorded as zero, one or two (both). Any birds which underwent emergency euthanasia as a result of a failed kill were excluded from post-mortem data since their anatomical damage was confounded by the emergency MCD.

At the end of each killing method assessment, a short questionnaire was completed by each stockworker. This consisted of three questions (1) whether they found the NMCD device helpful in dislocating birds’ necks; (2) whether they preferred the NMCD device over the MCD method; and (3) whether they would consider using the NMCD device as an on-farm killing method if it were made available, as a replacement for MCD.

### Statistical Analysis

Data were collected at the bird level and stockworker level and were summarized in Microsoft Excel (2010) spreadsheets and analyzed using Genstat (16th Edition). Statistical significance was termed by a threshold of 5% probability based on F tests. Summary graphs and statistics were produced at the stockworker level. For all models the random effects included the stockworker. All fixed effects were treated as factors and classed as categorical classifications. 

Generalised Linear Mixed Models (GLMMs) using logit link function and binomially distributed errors due to the nature of the binary data were used to statistically compare kill efficacy and post-mortem parameters across stockworkers. In the maximal models, fixed effects included killing method, bird type, training level, bird order, session, handedness, technique, and all their interactions. Co-variates included bird weight and neck gap size. As kill performance was dependent on number of kill attempts as well as generating a gap between two cervical vertebra, birds which were scored as “no” for kill were not excluded from GLMM analysis of post-mortem measures, but instead kill performance was incorporated as a factor into the model. Logit link function and binomially distributed errors were used due to the nature of the binary and categorical data, in order to compare post-mortem measures and their consistency across stockworkers in successful kills only, as post-mortem assessments of unsuccessful kills were invalidated by confounding damage by emergency or multiple attempts. For the size of neck gap variable, distribution was normal and the logit link function not used. Dispersion was fixed dependent on the variable. 

Frequency differences in the binomial (yes/no) questionnaire data for the answers to all three questions across the eight stockworkers were analyzed using Chi-Square tests, with the expected observations assumed to be “no” as the NMCD device had not be used or seen by the stockworkers prior to the trial. Further statistical analysis of stockworkers sub-divided by bird type were not undertaken due to low sample size.

## 3. Results

Variation between stockworker biometric measures was minimal with handedness evenly split across the eight stockworkers. There was a bias towards left handedness (3/4 stockworkers) on the broiler farm and right handedness on the layer farm (3/4 stockworkers). All sizes of NMCD were chosen and used by the stockworkers, despite minimal hand size variation, with the majority of stockworkers choosing the “Large” sized glove (5/8 stockworkers). One broiler and all 4 layer stockworkers used the “V” technique and 3 broiler stockworkers used the “R” technique for dislocating chickens’ necks. Therefore, the two techniques were bird type specific, with only broiler stockworkers using the “R” technique.

### 3.1. Killing Performance

Individual stockworker performance is summarized in Table 1. Mean stockworker kill performance was significantly higher for the MCD (98.4 ± 0.5%) killing method compared to NMCD (81.6 ± 1.8%) (*p* < 0.001). Two stock workers from the broiler farm opted not to continue onto live bird tests for NMCD. MCD had a lower mean and maximum number of kill attempts (mean = 1.0 ± 0.0, max = 2) compared to NMCD (mean = 1.3 ± 0.0, max = 3). 

Bird type had an effect on kill performance, irrespective of killing method, with a better mean kill performance in layer hens (88.4 ± 7.5%) compared to broilers (81.5 ± 12.3%) (*p* = 0.041). There was also an interaction between killing method and bird type (*p* = 0.035); with laying hen kill performance being higher (80.0 ± 14.2%) compared to broilers (63.1 ± 21.9%) with NMCD. The opposite interaction was apparent with MCD (broilers = 100.0 ± 0.0%; layer hens = 96.8 ± 1.6%) (*p* = 0.035). The training level required for NMCD had a significant effect on kill performance (*p* = 0.038) with the second training associated with a lower kill performance. There were no other interactions between other factors and training level (e.g., time of day, bird type, etc.). There was a significant interaction between training level required and technique (“V” or “R”, *p* = 0.017) with stockworkers who used the “R” technique requiring more training (training level mean = 1.6 ± 0.4) than stockworkers who used the “V” technique (training level mean = 1.2 ± 0.2). Handedness and the interaction between killing method and handedness had no effect on killing performance.

Bird order had an effect on kill performance (*p* = 0.003), with lower kill success being associated with birds killed early in the test (mean bird order = 16.2 ± 3.1) compared to birds killed later (mean bird order = 37.8 ± 4.6) regardless of method. A significant interaction between killing method and bird order also demonstrated that MCD kill performance decreased as birds were killed (*p* = 0.009) (mean kill order for: failed kill = 48.3 ± 2.2; and successful kill = 33.1 ± 4.7), while the opposite effect was seen for NMCD, where performance improved (mean kill order for: failed kill = 14.8 ± 3.6; and successful kill = 35.7 ± 3.8). Session (morning/afternoon) also had an effect (*p* = 0.018), with kill performance improving in the afternoon (mean 1.0 ± 0.0) compared to the morning (mean 0.9 ± 0.0) (*p* = 0.022). In particular, NMCD, kill performance was better in the afternoon session compared to the morning (morning: 0.7 ± 0.0; afternoon: 0.9 ± 0.0). There was no effect of day. 

### 3.2. Post-Mortem Parameters

The calculated means (±SE) for the selected post-mortem parameters, at the stockworker level, are shown in Table 2. The remaining binary measures (yes/no) had no variation across stockworkers or killing method, achieving 100% cervical dislocation, subcutaneous hematoma and spinal cord severed in all successfully killed birds. There was also no variation in vertebral damage with all stockworkers causing none. For all other post-mortem measures, the NMCD method was more likely to sever carotid arteries, achieve a higher dislocation level (e.g., C0–C1), and cause a larger neck gap size compared to MCD (Table 2). Broken skin and external bleeding was more likely with NMCD (Table 2). 

**Figure 2:**
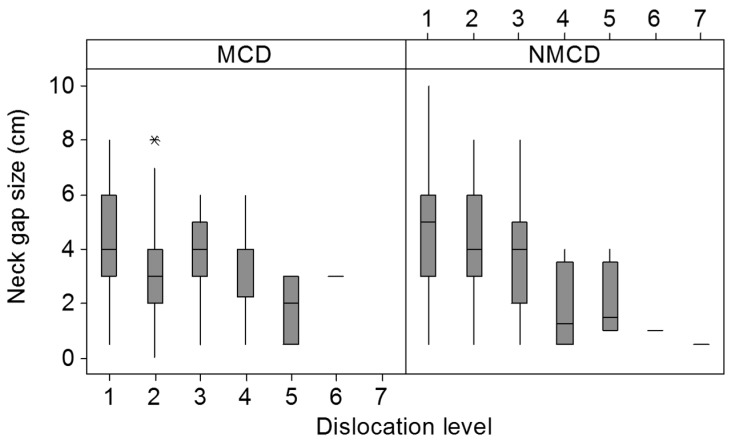
Effect of neck gap size (cm) on dislocation level (where 1 = between vertebrae C0–C1, etc.) for both killing methods. The asterisk (*) indicates an outlier.

Dislocation level was not affected by whether the kill was successful, bird order, or the interaction between killing method and bird order. Bird type had an effect on dislocation level with higher dislocations (e.g., C0–C1) more likely to occur in broilers (mean = 1.4 ± 0.0) than in layers (mean = 1.7 ± 0.0). Training level also had an effect on NMCD (*p* = 0.042) with the highest dislocation levels (closest to the skull) achieved at training level 2 (mean 1.2 ± 0.0) compared to level 1 (mean 1.6 ± 0.0). Lower dislocations were significantly more likely to occur in morning sessions (mean 1.4 ± 0.0) compared to afternoon sessions (mean 1.6 ± 0.0) (*p* < 0.001). Bird weight also had an effect on dislocation level (*p* = 0.042), with lower dislocations occurring in lighter birds (*r* = −0.243, *p* = 0.032). Neck gap size (*p* < 0.001) and the interaction between it and killing method (*p* = 0.038) had an effect on dislocation level, with larger neck gap sizes occurring with higher dislocation levels, with NMCD producing larger neck gap sizes for high dislocation levels compared to MCD (Figure 2).

**Figure 3:**
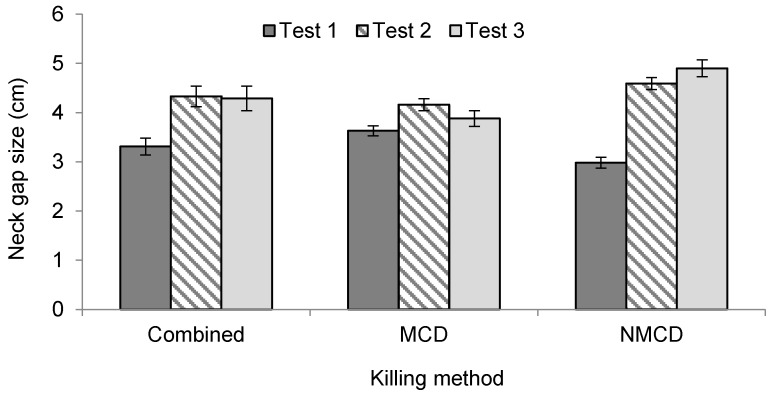
Effect of test number (*p* < 0.001) on neck gap size for the killing methods combined, as well as the individual killing methods.

**Figure 4:**
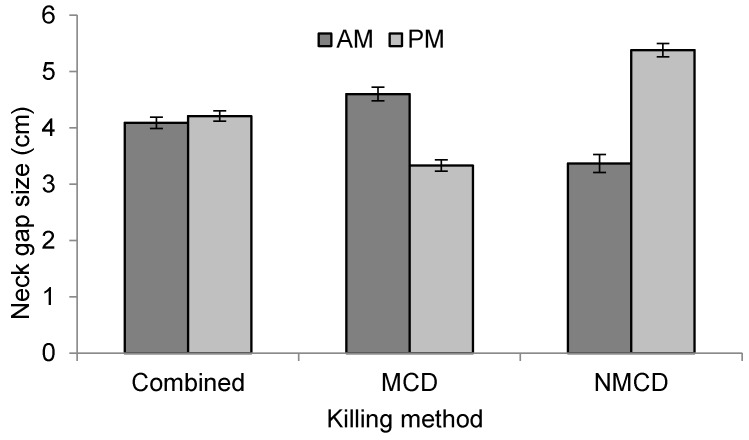
Effect of session (morning (AM) or afternoon (PM), (*p* < 0.001)) on neck gap size for combined and individual killing methods.

Neck gap size was not affected by kill success or number of kill attempts, but laying hens exhibited larger neck gap sizes (mean = 4.7 ± 0.1 cm) than broilers (mean = 3.5 ± 0.1 cm) (*p* = 0.039). With NMCD, training also had an effect on neck gap size (*p* < 0.001), with the larger neck gap sizes seen at training level 2 (mean = 5.8 ± 0.2 cm) compared to level 1 (mean = 4.3 ± 0.1 cm). Bird number (*p* = 0.002) was positively correlated (*r* = 0.10, *p* = 0.002) with neck gap size. Test stage also had an effect (*p* < 0.001), as did the interaction with killing method (*p* < 0.001), demonstrating that neck gap size increased with test stage overall, however the interaction with killing method demonstrated that MCD was not associated with an increase in neck gap size with test stage (Figure 3). Session had an effect on neck gap size (*p* < 0.001), as did its interaction with killing method (*p* < 0.001) (Figure 4), which showed that overall, neck gap size was slightly larger in the afternoon session regardless of stockworker. However when incorporating killing method, MCD showed a decrease in neck gap size during the afternoon session compared to the morning, with the opposite relationship for NMCD.

The NMCD method was more likely to sever a minimum of one artery compared to the MCD method (Table 2). Whether the kill was successful or not had no effect on whether a carotid artery was severed, and neither did bird type. Bird order also had no effect, however when taking into account the killing method, the number of carotid arteries severed was higher for birds killed nearer the start of the test for MCD, while there was no such effect with NMCD (*p* < 0.001). There was also a significant interaction between killing method and session (*p* = 0.032), with stockworkers who performed in the morning for MCD more likely to severe a carotid artery than those in the afternoon (AM = 0.2 ± 0.1; PM = 0.1 ± 0.0), but there was no effect for NMCD (AM = 0.5 ± 0.1; PM = 0.5 ± 0.1). Bird weight, test number, dislocation level, or number of kill attempts had no effect on the number of carotid arteries severed. For NMCD tests, training level had an effect (*p* = 0.014), with training level 2 showing the highest mean number of carotid arteries severed (mean = 0.8 ± 0.1) compared to level 1 (mean = 0.5 ± 0.0). Neck gap size as a factor had an effect on the number of carotid arteries severed (*p* < 0.001), with a positive correlation (*r* = 0.483, *p* < 0.001), but there was no interaction with killing method.

Whether or not the skin was broken was affected by kill success (*p* < 0.001), with the skin more likely to be broken in unsuccessful kills (kill success: yes = 1.6% skin broken; no = 20.7% skin broken). The number of kill attempts had an effect (*p* < 0.001) on likelihood of skin tears, with the greater number of kill attempts being positively associated with the percentage of birds with skin tears (*r* = 0.388, *p* < 0.001). There was no interaction between number of kill attempts and killing method. Bird weight had no effect on whether or not the skin was torn during application of either method.

### 3.3. Questionnaire

The percentage of stockworkers which answered yes to each question is shown in Table 3. Chi-Square tests showed that there were significant differences between the expected and observed counts for each question (Q1, Q2, Q3) across the eight stockworkers (Q1 χ^2^ = 107.65, *p* < 0.001; Q2 χ^2^ = 46.54, *p* < 0.001; Q3 χ^2^ = 194.02, *p* < 0.001).

## 4. Discussion

Evaluation of user reliability and consistency of a novel killing device in its intended environment is a vital part of its detailed assessment. The NMCD device was previously evaluated in laboratory environments with one user, where it was demonstrated to produce a high kill success rate and to increase the trauma to the neck (e.g., severing of carotid arteries) compared to MCD and other novel captive bolt and cervical dislocation devices [12,13,22]. In the current study, we have extended this work to provide a practically relevant comparison between the NMCD and standard MCD on laying hen and broiler farms, with four stockworkers per farm. Evaluation of the methods in this commercially-relevant context demonstrated that the NMCD device was not as reliable as MCD for killing poultry in a commercial setting and across multiple users, based on the number of kill attempts and immediate behavioral sign of brain death (cessation of rhythmic breathing). 

It is worth noting that there was substantial variation between stockworkers in kill performance, and despite NMCD being designed around the MCD method, our findings suggest that a reliable kill performance with MCD did not guarantee a reliable kill performance with NMCD. However, as in the lab, the NMCD method did increase the consistency and amount of anatomical trauma to birds’ necks. Such damage has previously been linked to loss of unconsciousness and brain death [31,32,33,34,35], suggesting that NMCD has at least the potential to cause reduced latencies to unconsciousness and brain death compared to MCD. 

During the NMCD tests, there was an apparent advantage for stockworkers who performed in the afternoon compared to the morning, and this is potentially due to observations of the use of the device during morning sessions when they were assisting. Post-mortem measures showed that stockworkers who performed the NMCD treatment in the afternoon had better performances than stockworkers who performed in the morning, with higher mean dislocation levels, larger neck gap sizes and higher likelihood of one or two carotid arteries being severed. This apparent effect of prior exposure to the method suggest that more extensive training and observation prior to using NMCD would be beneficial. There was no afternoon advantage for stockworkers when using MCD, instead a slight disadvantage was apparent, with afternoon performances being associated with a marginal decrease in kill efficacy. This could be attributed to fatigue (physical and/or mental), as those stockworkers had also assisted in the morning. This apparent fatigue affect could have been reduced in the NMCD performance, as the glove provided support to the hand and wrist [12,13].

All of the stockworkers were experienced in MCD and had been approved as competent by their farm managers, so the MCD treatment had an expected advantage of prior experience compared to NMCD. An unexpected hurdle for some of the stockworkers was adapting to the NMCD treatment, especially when their MCD method was not the standard “V” technique [3], but the “R” technique instead. The NMCD device is designed around the “V” technique, and so probably provides users who perform MCD in this way with an advantage. This notion was supported by the increased NMCD training required for stockworkers who used the “R” technique. Interestingly, the technique employed was bird type specific, with only broiler stockworkers using the “R” technique. This is likely to be a result of the training they received as part of their on-farm standard operating procedures. Stockworkers which used the “R” technique had 100% kill success in MCD, but the post-mortem results demonstrated it produced the less severe trauma to the neck compared to the “V” MCD technique, and the NMCD treatment. It was also observed that on some occasions (27/1000 birds) some of the stockworkers adopted a ‘double pull’ technique when using either treatment, although the mostly in the NMCD treatment. This automatically resulted in recording of a bird kill failure, as it was defined as two attempts. The double pull appeared to be a somewhat unconscious mechanism by the stockworker to “double-check” the dislocation had occurred, with the pulls occurring in rapid succession. This resulted in the death of the bird, however was deemed an application failure. There is no way of knowing whether the first pull resulted in a complete dislocation and whether the second rapid pull (or stretch) was a welfare concern. However, it is a concern that the stockworkers felt they had to perform the double pull, perhaps because they were not confident with their application and birds may not have not been fully cervically dislocated on the first attempt, with the risk that they experienced pain [7,9,11,36,37,38]. 

In the NMCD treatment the majority of stockworkers only required training level one suggesting the device was fairly intuitive to use following the reading of the leaflet. Three stockworkers required further training, one was a laying hen stockworker and the other two worked with broilers. Both of the broiler stockworkers used the “R” technique for MCD and reached the maximum training level. One stockworker chose not to continue due to continued unease with using the device, while the other stockworkers testing was discontinued on welfare grounds, as there was concern that this individual was not concentrating or complying with the training correctly. Based on this small sample size, the results suggest that the training levels provided here were not sufficient to train stockworkers who did not use the “V” technique and therefore may not be sufficient to train “V” inexperienced people to use the NMCD method. However, note that training individuals not experienced in MCD was not the aim of this study. While the levels of training employed were not sufficient to fully re-train individuals to a different method, for the majority of stockworkers irrespective of training level, kill performance and anatomical trauma to the birds’ necks (e.g., neck gap size, number of carotid arteries, dislocation level) increased during the NMCD tests for each stockworker, suggesting performance improved with practice. The opposite effect was seen with MCD, where stockworkers appeared to slightly decrease their kill performance over time despite stockworker being trained in that method and deemed competent, highlighting the concern that MCD is not a consistent method, irrespective of training or experience [1,30]. This inconsistency could be due to slight variations in method application, stockworker fatigue (including hand/arm muscle fatigue), and/or lack of concentration over time (i.e., boredom). 

There was a general trend that kill performance was lower in Test 1 (cadavers), irrespective of killing method, which could be attributed to the apparent difficulty in performing cervical dislocation on birds when there was no resistance due to a lack of muscle tone. This is likely to make it difficult to ascertain when the dislocation has occurred and this also explains the slightly higher number of kill attempts (i.e., multiple pulls/stretches) and over-stretching (i.e., accidental decapitations) in Test 1 compared to other tests across both killing methods. These results suggest that using cadavers as a training aid for MCD or NMCD may be of limited value.

Unlike in previous work [13], the kill success rate in broilers was lower compared to laying hens; however this could also be an artefact of the “R” technique, which 3/4 of broiler stockworkers used, as well as two of these stockworkers not completing the live bird tests, therefore reducing their overall kill performance. There was considerable variation across stockworkers in terms of their consistency of anatomical trauma produced as a result of each killing method. The NMCD method did not reduce the likelihood of intra-vertebral damage, or improve the likelihood of a dislocation occurring, or the spinal cord being severed. Therefore, NMCD did not outperform MCD for these measures. More than 58% of all birds, irrespective of killing method, received a C0–C1 dislocation level, although birds killed by NMCD were more likely to receive a C0–C1 dislocation method (MCD = 58.6%; NMCD = 69.1%). This focused the anatomical damage to the top of the spinal cord and possibly the base of brain stem. Damage to this area is associated with spinal cord concussion, neurogenic shock and loss of consciousness, suggesting NMCD was more likely to result in birds’ losing consciousness post application than MCD [32,33,35,39,40,41]. 

Bird weight was negatively correlated with dislocation level, which is the opposite effect to that seen in previous work [7,11,13,30,33]. In the current study, heavier birds were more difficult to cervically dislocate at C0–C1 compared to lighter birds. Larger neck gap sizes were associated with higher dislocation levels, which suggests higher potential trauma. Once the dislocation had been achieved the “follow-through” stretch (2nd stage of technique: “twist and stretch” [3]), which causes the neck gap, demonstrates the ease in dislocating and stretching the birds’ necks, therefore smaller neck gap sizes could be attributed to difficulty in causing the dislocation which may have limited the “follow-through” stretch. The C0–C1 connection is heavily protected and reinforced by connective tissue and is the join between the skull (occipital condyle) and the top of the spine, with C1 being the smallest cervical vertebra [7,9,42]. This makes dislocation between C0–C1 very difficult compared to inter-vertebral dislocation between similar sized and shaped vertebrae. As the number of carotid arteries severed is highly associated with neck gap size, it can be suggested that the “follow-through” stretch is paramount to causing the severing of one or more of the carotid arteries, and therefore reducing the blood supply to the brain and causing cerebral ischemia [11,35,43].

In terms of biosecurity and practicality, less external blood loss and skin tears are preferred in commercial environments [44], as well as being aesthetically more appealing. The likelihood of skin tearing was higher in unsuccessful kills, mainly attributed to the higher number of kill attempts and greater risk of over-stretching the neck. Interestingly, bird type or the confounded MCD method (“R” technique) was more likely to be related to skin tearing, and was more likely to result in a lower dislocation level and fewer carotid arteries severed, suggesting that the “R” technique consistently performed sub-optimally in comparison to the “V” technique, as well as the NMCD treatment. For both methods, skin tearing occurred more in cadavers than in live birds, again highlighting the difficultly in performing the treatments on a bird which has no muscle tone, and perhaps the lack of usefulness of cadaver practice for training.

The questionnaire revealed that less than half of stockworkers (3/8) considered the NMCD device a useful aid for dislocating bird’s necks, and only two stockworkers stated they preferred the device to MCD. Despite this, 50% of stockworkers stated they would consider using the NMCD device as an alternative to the now restricted MCD method, irrespective of their currently used MCD approach (“V” or “R” technique). Surprisingly, the stockworkers who said they would consider using the NMCD device did not have the highest performance rates in the method, but instead were the worst performers, but did perform well in MCD. The interpretation of this is difficult to determine; these stockworkers may be willing to consider an alternative that is similar to MCD in preference to other alternative methods (e.g., Cash Poultry Killer). Since only half of the stockworkers would consider it, this study demonstrates that a strong preference for MCD was present and the limited practice with the NMCD provided in this study was not sufficient to encourage stockworkers to use it in place of MCD.

## 5. Conclusions

In conclusion, NMCD did not match the kill performance of MCD in a commercially relevant environment and did not completely remove variation in kill success and trauma generated by various users. However, the NMCD device was more likely to perform optimally when it was successful (e.g., severing carotid arteries, achieving C0–C1 dislocation) compared to MCD, suggesting it has promise if training could be optimized. It appears to be particularly problematic to train stockworkers to use NMCD when their usual MCD method was the “R” technique, and as the scale of use of this technique in the poultry industry is currently unknown, it is difficult to judge the effect this may have on uptake and successful use of the NMCD method. The basic training provided for NMCD (a leaflet) was adequate for some stockworkers, and the performance of all stockworkers improved with practice. Barriers remain in terms of the willingness of stockworkers to consider an alternative method to standard MCD, even if their feedback from the trial’s questionnaire showed potential. Collectively, the results indicate that instruction to use the NMCD method requires further refinement and perhaps two training approaches would be most appropriate: one targeted at “V” technique experienced individuals and another aimed at completely inexperienced individuals (including “R” technique users). Such tailored instruction would be likely to incentivize use of NMCD and optimize its performance in terms of bird welfare outcomes. 

## Figures and Tables

**Table 1:** Stockworker performance, training required and agreement to each test for both killing methods, sub-divided by bird type.

Killing Method	Bird Type	Stockworker	Training Stage	Successful Kill Performance		
				Test 1	Test 2	Test 3
MCD	Broiler	1	-	10/10	30/30	30/30
		2	-	10/10	30/30	30/30
		3	-	10/10	30/30	30/30
		4	-	10/10	30/30	30/30
	Layer	5	-	10/10	30/30	29/30
		6	-	10/10	28/30	27/30
		7	-	10/10	30/30	30/30
		8	-	9/10	30/30	28/30
NMCD	Broiler	1	1	7/10	27/30	30/30
		2	2	9/10	*	*
		3	1	10/10	29/30	27/30
		4	2	0/10	*	*
	Layer	5	1	10/10	30/30	30/30
		6	2	0/10	9/30	18/30
		7	1	10/10	30/30	28/30
		8	1	2/10	30/30	27/30

* Stock workers declined to continue the test.

**Table 2:** Descriptive statistics (mean, SE, minimum, and maximum) as well the GLMM results (where possible) for comparison of all post-mortem measures by killing method.

Post-Mortem Measure	Killing Method	Mean	SE	Min.	Max.	*p* Value
Number of carotid arteries severed	MCD	0.1	0.0	0.0	2.0	<0.001
	NMCD	0.5	0.0	0.0	2.0	
Dislocation occurred ^+^	MCD	2.0	0.0	2.0	2.0	
	NMCD	2.0	0.0	2.0	2.0	
Dislocation level *	MCD	1.6	0.0	1.0	6.0	0.002
	NMCD	1.4	0.0	1.0	7.0	
External bleeding ^+^	MCD	1.0	0.0	1.0	2.0	<0.001
	NMCD	1.2	0.0	1.0	2.0	
Neck gap size (cm)	MCD	3.9	0.1	0.0	8.0	<0.001
	NMCD	4.4	0.1	0.0	10.0	
Skin broken ^+^	MCD	1.0	0.0	0.0	2.0	<0.001
	NMCD	1.2	0.0	0.0	2.0	
Spinal cord severed ^+^	MCD	2.0	0.0	2.0	2.0	
	NMCD	2.0	0.0	2.0	2.0	
Subcutaneous hematoma ^+^	MCD	2.0	0.0	2.0	2.0	
	NMCD	2.0	0.0	2.0	2.0	
Vertebral damage ^+^	MCD	1.0	0.0	1.0	1.0	
	NMCD	1.0	0.0	1.0	1.0	

* Dislocation point means were calculated by converting vertebral levels to a numerical category (e.g., C0–C1 = 1; C1–C2 = 2; C2–C3 = 3; etc.). ^+^ Binary yes/no means were calculated by conversion to numerical categories (e.g., no = 1; yes = 2).

**Table 3:** Proportions of agreement (answered ‘yes’) for the three questions asked to stockworkers which worked with broilers; and laying hens following NMCD tests.

Question	Broiler Stockworkers	Layer Stockworkers
(1) Was NMCD helpful?	1/4 (25%)	2/4 (50%)
(2) Prefer NMCD to MCD?	0/4 (0%)	2/4 (50%)
(3) If available, would you use NMCD?	2/4 (50%)	2/4 (50%)
